# Oral Curcumin (Meriva) Is Effective as an Adjuvant Treatment and Is Able to Reduce IL-22 Serum Levels in Patients with Psoriasis Vulgaris

**DOI:** 10.1155/2015/283634

**Published:** 2015-05-18

**Authors:** Emiliano Antiga, Veronica Bonciolini, Walter Volpi, Elena Del Bianco, Marzia Caproni

**Affiliations:** Section of Dermatology, Department of Surgery and Translational Medicine, University of Florence, Viale Michelangiolo 41, 50125 Florence, Italy

## Abstract

Curcumin is a complementary therapy that may be helpful for the treatment of psoriasis due to its anti-inflammatory, antiangiogenic, antioxidant, and antiproliferative effects. In the present study we performed a randomized, double-blind, placebo-controlled clinical trial to assess the effectiveness of a bioavailable oral curcumin in the treatment of psoriasis. Sixty-three patients with mild-to-moderate psoriasis vulgaris (PASI < 10) were randomly divided into two groups treated with topical steroids and Meriva, a commercially available lecithin based delivery system of curcumin, at 2 g per day (arm 1), or with topical steroids alone (arm 2), both for 12 weeks. At the beginning (T0) and at the end of the therapy (T12), clinical assessment and immunoenzymatic analysis of the serum levels of IL-17 and IL-22 were performed. At T12, both groups achieved a significant reduction of PASI values that, however, was higher in patients treated with both topical steroids and oral curcumin than in patients treated only with topical steroids. Moreover, IL-22 serum levels were significantly reduced in patients treated with oral curcumin. In conclusion, curcumin was demonstrated to be effective as an adjuvant therapy for the treatment of psoriasis vulgaris and to significantly reduce serum levels of IL-22.

## 1. Introduction

Psoriasis is a common chronic inflammatory disease of the skin, nails, and joints, which affects about 2% of the general population [1], with a significant impact on long-term quality of life [2]. The potential organ toxicity associated with chronically used systemic drugs, their immunosuppressive effects with the increased risk of infections and malignancies, and the high costs of some of them justify a novel assessment of therapeutic targets and modalities in patients with psoriasis [3, 4].

An estimated 80% of individuals in developing countries depend primarily on natural products to meet their healthcare needs, and recent surveys suggest that one in three Americans also uses medicinal natural products daily [5]. With regard to psoriasis, 51% of the patients use complementary and alternative medicine therapies to treat their skin, despite limited or no scientific data on the safety and efficacy of these treatments [6]. Among them, curcumin (dihydroferuloyl-methane, the active component of the Indian spice turmeric) has been used for centuries in traditional medicines of China and India [7], paving the way for the development of several studies both in cultured cells and in animal models, which revealed a surprisingly wide range of beneficial properties of this yellow-pigmented spice, including anticancer and anti-inflammatory activity [8–12]. Furthermore, the extremely good safety profile of curcumin represents the most compelling and key rationale for its use, as to date no studies have discovered any relevant toxic effects even at very high doses [13, 14]. Whereas curcumin has several molecular targets, it may be promising for the treatment of psoriasis interacting with the main pathogenetic pathways of the disease, namely, T cell-mediated inflammation via the inhibition of nuclear factor kappa B (NF-*κ*B) [15–17], keratinocyte proliferation, inhibiting phosphorylase kinase (PhK) [18–20], and angiogenesis [21–23].

The use of curcumin in the treatment of psoriasis is essentially based on anecdotal reports; however, few previous studies investigated its efficacy in psoriasis patients. In particular, Heng et al. [24] tested the effect of topical curcumin in 10 patients with psoriasis. Interestingly, topical curcumin was able to improve psoriasis lesions and to inhibit PhK activity, supporting the hypothesis that effective antipsoriatic activity may be achieved through the modulation of keratinocyte proliferation. In another study [25], the oral formulation of curcumin has been tested in patients with moderate to severe psoriasis vulgaris, with a low overall response rate.

In this study, we used Meriva, a novel bioavailable lecithin based delivery form of curcumin [26] as an adjuvant therapy for the treatment of patients with mild-to-moderate psoriasis vulgaris. In particular, we compared the efficacy of topical methylprednisolone aceponate 0.1% plus oral curcumin versus topical methylprednisolone aceponate 0.1% plus oral matching placebo. Moreover, we investigated the effects of such a treatment on the serum levels of IL-17 and IL-22 that represent the prototypical cytokines secreted by T helper 17 (Th17) and T helper 22 (Th22) cells, respectively, and are thought to play a major role in the pathogenesis of the disease [27–33].

## 2. Materials and Methods

### 2.1. Study Population

Patients having mild-to-moderate plaque psoriasis histologically confirmed with a Psoriasis Area Severity Index (PASI) < 10 were included into the study. The exclusion criteria were the following: patients < 18 years old; patients with guttate, erythrodermic, or pustular psoriasis, as well as with psoriatic arthritis; patients who used systemic treatments for psoriasis within 3 months before day 0 or at any time during the study; patients who used topical treatments or phototherapy for psoriasis in the month before the study or at any time during the study; pregnant or lactating women, or women of childbearing age not using a reliable method of contraception; patients with clinically significant laboratory abnormalities at screening, or significant uncontrolled comorbidities. Enrolled participants were required to avoid prolonged exposure to sun or UV light and to discontinue nonmedicated emollients and medicated psoriasis shampoos 24 hours before each study visit. The study was conducted in accordance with the Declaration of Helsinki and was approved by the institutional review board of the Hospital Ospedale Piero Palagi (Florence, Italy); all patients gave written informed consent.

### 2.2. Study Product

Patients were instructed to take 2 tablets 2 times a day, each tablet containing 500 mg Meriva (Indena SpA, Milan), for an overall daily dose of 2 g.

Meriva is a patented delivery form of the dietary phenolic curcumin, resorting to the use of lecithin (from non-GMO sources) to boost the absorption and bioavailability. The two ingredients form a noncovalent adduct in a 1 : 2 weight ratio, and two parts of microcrystalline cellulose are then added to improve flowability, with an overall content of curcumin of 20% (e.g., 100 mg for each tablet).

### 2.3. Study Design

This was a phase III, single-dose, randomized, double-blind, placebo-controlled clinical trial in patients with chronic plaque psoriasis. The study was performed at the Department of Surgery and Translational Medicine, Section of Dermatology (University of Florence, Italy), and consisted in a screening period, a baseline time in which PASI score was assessed and the patients started the treatment, a treatment period of 12 weeks, and a 4-week follow-up period. Patients were randomly assigned in a 1 : 1 ratio to receive topical methylprednisolone aceponate 0.1% ointment (once daily on the lesions) plus 2 g per day of Meriva (2 tablets of 500 mg, twice daily) (arm 1) or topical methylprednisolone aceponate 0.1% ointment (once daily on the lesions) plus matching placebo (2 tablets identical for size, form, and color from the verum, twice daily) (arm 2).

Patients were seen at a screening visit and then at baseline (T0) and weeks 4, 8, 12 (T12), and 16 (T16) for safety and efficacy evaluations. At T0 and at T12, serum samples were collected to investigate the levels of IL-17 and IL-22. The first participant was enrolled on May 3, 2012, and all study procedures were concluded on May 27, 2014.

### 2.4. Efficacy Endpoints

The primary objective of the study was to assess the superiority of topical methylprednisolone aceponate plus Meriva (arm 1) over topical methylprednisolone aceponate plus placebo (arm 2), evaluating the reduction of disease activity calculated by PASI (a composite evaluation instrument for psoriasis severity, with subscores for erythema, induration, scaling, and percentage of body-surface area affected) in both arms after 12 and 16 weeks from baseline. Secondary efficacy endpoints included PASI 50, PASI 75, PASI 90, and PASI 100 (reductions of 50%, 75%, 90%, and 100%, resp., in the baseline PASI score) that have been shown to represent a meaningful endpoint in psoriasis clinical trials [34]. Each endpoint was assessed by means of a between-group comparison of the proportion of patients who met the criterion for that objective.

### 2.5. Safety Endpoints

All patients who received at least one dose of study product were included in the safety analysis. Safety data were obtained by patient interview and the clinical examination conducted by the physician at all study visits.

### 2.6. Cytokine Analysis

Blood samples were collected from patients of both groups at baseline and T12 and were centrifuged for 20 min at 10,000 ×g (5417R microcentrifuge; Eppendorf, Hamburg, Germany). Then, serum samples were subdivided into small aliquots to be stored at −80°C until tested for cytokine levels. Solid-phase enzyme-linked immunosorbent assay (ELISA) kits were used to determine IL-17 and IL-22 serum levels (R&D Systems, Minneapolis, MN, USA), according to the manufacturer's instructions. Results are expressed in picogram per milliliter (pg/mL) as mean ± standard deviation. Samples and standards were analyzed in duplicate and only variation coefficients <15% were accepted.

### 2.7. Statistical Analysis

Statistical analysis of clinical efficacy of curcumin was performed with a Wilcoxon signed rank test between the pretreatment and posttreatment groups, and a Wilcoxon rank sum test was used for comparing arm 1 with arm 2. The serological data of IL-17 and IL-22 were analyzed with Student's *t*-test. In order to verify the homogeneity of the two groups, we preliminarily compared clinical and serological parameters between the two pretreated series. Differences were considered as statistically significant when *P* < 0.05.

## 3. Results

**Figure 1:**
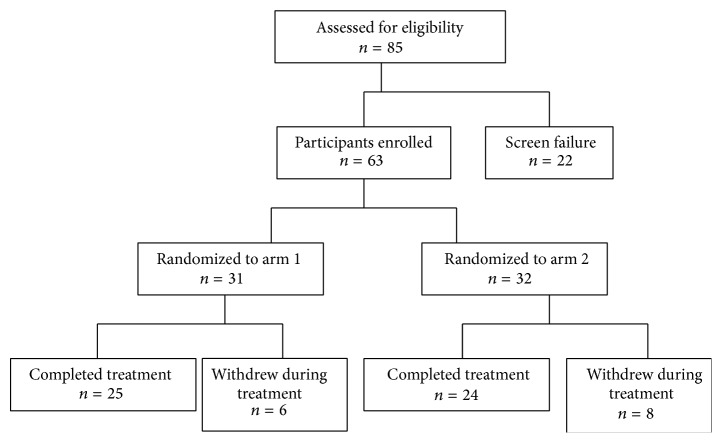
Flowchart of participant enrollment.

A total of 85 patients were screened, 63 of whom were enrolled and were randomized in one of the two arms (Figure 1). Of the 22 participants who were not included in the study, 4 were excluded because they had a PASI > 10, 9 had used topical treatments within 30 days before day 0, 5 had used systemic treatments within 3 months before day 0, and 4 had concurrent arthropathy. Baseline demographic information of the participants is reported in Table 1.

Thirty-one patients were randomized into arm 1, while 32 into were randomized arm 2. Of the 31 patients enrolled into arm 1, 25 completed the trial up to week 16. Six out of 31 enrolled participants into arm 1 did not complete the trial. Two were withdrawn by the investigators before week 12 because of lack of efficacy and one for side effects (diarrhoea) and 3 were lost to follow-up.

Of the 32 patients into arm 2, 24 completed the trial up to week 16. Eight out of 32 enrolled participants did not complete the trial. Six were withdrawn by the investigators before week 12 because of lack of efficacy and/or worsening of their psoriasis and two for side effects (papular eruption on the face and nausea, resp.).

### 3.1. Efficacy Endpoints

**Figure 2:**
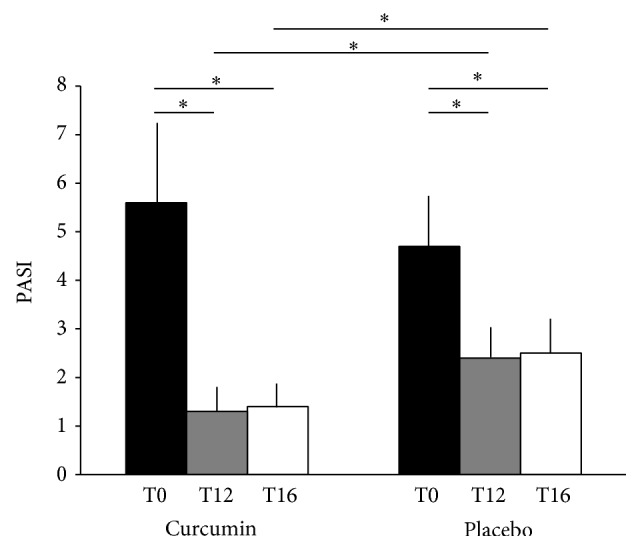
Median PASI values [25°–75° percentile] at baseline (T0) and after 12 weeks (T12) in patients treated with topical methylprednisolone + Meriva (curcumin) or with topical methylprednisolone + placebo (placebo). ^∗^ *P* < 0.05.

Median PASI values [25th–75th percentile] at T0 were 5.6 [4.2–7.3] for arm 1 and 4.7 [3.8–5.8] for arm 2. At T12, both groups achieved a significant reduction of PASI values (arm 1: PASI at T12 = 1.3 [0.6–1.7], PASI T0 versus PASI T12: *P* < 0.05; arm 2: PASI at T12 = 2.4 [1.4–3.0], PASI T0 versus PASI T12: *P* < 0.05; Figure 2). However, the reduction of PASI values was higher in patients treated with both topical steroids and Meriva (arm 1) than in patients treated with topical steroids plus placebo (arm 2) (*P* < 0.05). The reduction of PASI remained significant for both groups at T16 (arm 1: PASI at T16 = 1.4 [1.2–1.8], PASI T0 versus PASI T16: *P* < 0.05; arm 2: PASI at T16 = 2.5 [1.8–3.3], PASI T0 versus PASI T16: *P* < 0.05; Figure 2). Moreover, at T16, patients included in arm 1 still had a more significant reduction in PASI values versus those included in arm 2 (*P* < 0.05; Figure 2).

Regarding the secondary efficacy endpoints, at T12, 23 patients in arm 1 (92%) and 13 in arm 2 (54%) achieved PASI 50, 12 patients in arm 1 (48%) and 3 in arm 2 (12%) achieved PASI 75, 5 patients in arm 1 (20%) and 1 in arm 2 (4%) achieved PASI 90, and 3 patients in arm 1 (12%) and 1 in arm 2 (4%) achieved PASI 100 (Table 2).

At the follow-up visit four weeks after the completion of the therapy, at T16, 22 patients in arm 1 (88%) and 11 in arm 2 (46%) still maintained PASI 50, 11 patients in arm 1 (44%) and 2 in arm 2 (8%) maintained PASI 75, 2 patients in arm 1 (8%) and none in arm 2 maintained PASI 90, and 1 patient in arm 1 (4%) and none in arm 2 maintained PASI 100 (Table 2).

### 3.2. Safety End Points

Only one out of 31 patients who received the study product and 2 out of 32 patients receiving placebo reported one adverse event. The patient treated with Meriva complained of diarrhoea for two days and decided to discontinue the study protocol after 8 weeks; diarrhoea was also associated with fever as well as to other symptoms of viral gastroenteritis; however it was not possible to assess the exact aetiology neither to attribute the adverse effect to the study product.

As far as the two patients administered matching placebo, one complained of a papular eruption on the face occurring after 5 weeks of therapy that was attributed to the study product by the patient who decided to retire from the trial. The other one suffered of nausea that occurred after 3 weeks and persisted for two weeks until the discontinuation of the therapy.

### 3.3. Serum Levels of Th17-Related Cytokines

**Figure 3:**
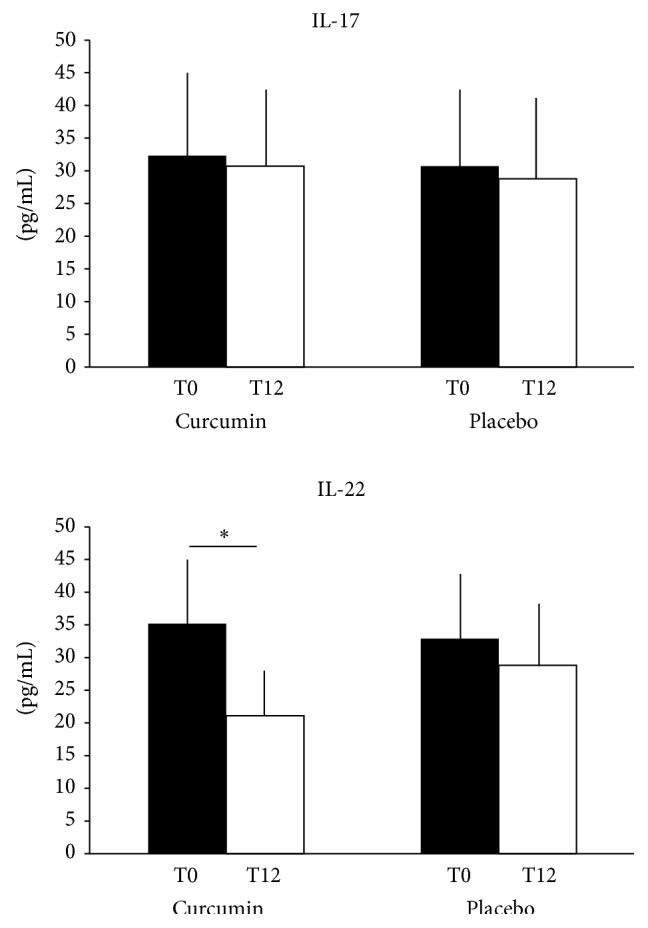
IL-17 and IL-22 mean values ± standard deviation at baseline (T0) and after 12 weeks (T12) in patients treated with topical methylprednisolone + Meriva (curcumin) or with topical methylprednisolone + placebo (placebo). Data are expressed as pg/mL. ^∗^ *P* < 0.05.

IL-17 and IL-22 were detected in all the patients. At T0, both groups showed similar IL-17 levels, without significant differences (32.3 ± 13.6 in group 1 and 30.7 ± 12.2 in group 2, resp.; Figure 3). After the treatment, at T12, no significant changes in IL-17 serum concentrations can be observed in both groups (30.7 ± 10.9 in group 1 and 28.8 ± 12.9 in group 2, resp.; Figure 3).

As for IL-17, IL-22 levels were similar in the two groups of patients with psoriasis at T0 (35.2 ± 9.5 in group 1 and 32.9 ± 11 in group 2, resp.; Figure 3). Interestingly, after 12 weeks, patients treated with topical methylprednisolone aceponate plus Meriva achieved a significant reduction of IL-22 serum levels (21.1 ± 7.5; IL-22 at T0 versus IL-22 at T12: *P* < 0.001), while the treatment with topical methylprednisolone aceponate plus placebo did not affect IL-22 expression (28.8 ± 10; Figure 3).

## 4. Discussion

The primary objective of our study was to investigate the efficacy of Meriva, a patented bioavailable formulation of curcumin, as an adjuvant therapy in patients with mild-to-moderate psoriasis vulgaris (PASI < 10) that are using topical steroids.

Our data demonstrated that the combination of methylprednisolone aceponate 0.1% ointment plus Meriva was superior to methylprednisolone aceponate 0.1% ointment plus placebo in treating psoriasis. In fact, although both regimens were able to significantly affect psoriasis lesions, patients using both topical steroid and Meriva showed a reduction of PASI values that was significantly higher than that of patients using topical steroid plus placebo. Moreover, the group treated with topical steroid and oral bioavailable curcumin achieved best PASI 50, PASI 75, PASI 90, and PASI 100 scores than patients treated with topical steroid plus placebo, suggesting that oral curcumin might be a useful adjuvant treatment for patients with psoriasis.

Moreover, oral curcumin was well tolerated in our study. In fact, only one patient discontinued the therapy due to adverse effects, namely, diarrhea, which was more likely associated with a viral gastroenteritis rather than with the drug itself. Accordingly, other studies confirmed the safety of curcumin in human beings, even at high doses [14], being mild gastrointestinal upset the most common complaint.

Our results are partially in contrast with those from a previous study, where curcumin was used alone for the treatment of patients with psoriasis [25]. In that study, the authors tested 4.5 g per day of oral curcuminoid C3 complex in 12 patients with psoriasis, of which only 8 completed the treatment protocol, with an intention-to-treat response rate of 16.7% and an as-treated response rate of 25% (considering responders the patients that achieved an improvement of their skin lesions >50%, as assessed by physician global assessment). However, the limited efficacy of curcumin in such a study could be related to the low number of patients enrolled in the trial or to the low bioavailability of the used oral curcumin formulation, probably secondary to its extensive reduction and conjugation in the intestinal tract [35, 36], as stated by the authors.

By contrast, the formulation of curcumin used in our study through the lipidic matrix can capitalize on the rapid exchange of phospholipids between biological membranes and the extracellular fluids, shuttling phenolics into biological membranes and increasing their cellular absorption.

In fact curcumin, as other dietary phenolics, is sparingly soluble both in water and in oily solvents but shows polar groups that can interact with the polar heads of phospholipids [37].

As a result, curcumin combination with phospholipids according to the Phytosome technology, as applied in Meriva, showed a marked increase in the concentration of plasma curcuminoids after oral administration in healthy volunteers [38], improving the clinical benefits at dosages significantly lower than those associated with uncomplexed curcumin.

As previously reported, curcumin can be effective in treating psoriasis by affecting several pathogenetic pathways. Moreover, recent studies performed in autoimmune models suggest that curcumin can modulate T helper immune responses, downregulating the Th1 and Th17 cell pathways [39, 40]. Accordingly, such activities might operate even in psoriasis. In fact, in a mouse model of imiquimod-induced psoriasis-like inflammation, topical curcumin was demonstrated to improve the skin lesions with an effect comparable to that of clobetasol and to significantly decrease the cutaneous levels of IL-17A, IL-17F, IL-22, IL-1*β*, IL-6, and TNF-*α* mRNA [41].

Interestingly, we observed that only the treatment with topical methylprednisolone aceponate 0.1% plus Meriva was able to significantly downregulate the concentrations of IL-22 in the serum of patients with psoriasis. As it is known, IL-22 is the prototypic cytokine secreted by Th22 cells, a subpopulation of T cells that selectively produces IL-22, but not IFN-*γ* nor IL-17. Several studies demonstrated that Th22 cells are increased in patients with psoriasis [28, 29, 42] and that IL-22 plays a major role in several steps of the pathogenesis of the disease, including inflammation and the proliferation of keratinocytes [43]. In fact, IL-22 induces keratinocytes to produce antimicrobial peptides, S100 acute-phase proteins, and chemokines (such as IL-8) and chemokine receptors, thus leading to neutrophilic chemotaxis within psoriasis lesions [44, 45]. Furthermore, IL-22 strongly induces hyperplasia of in vitro reconstituted human epidermis [45–47] and may be responsible for the acanthosis typically found in psoriasis. As a result, the effect on IL-22 and, therefore, on the Th22 pathway, may be included among the potential antipsoriatic activities of curcumin.

## 5. Conclusions

In conclusion, our study demonstrated that Meriva, a novel lecithin based delivery form of curcumin, can be regarded as a safe and effective ingredient, useful for the adjuvant therapy of patients with mild-to-moderate plaque psoriasis vulgaris treated with topical steroids. Among the several biological effects of curcumin, the ability to downregulate T cell-mediated inflammation, and in particular the Th22 pathway, may be considered one of the major mechanisms by which the product can control psoriasis.

## Figures and Tables

**Table 1:** Baseline demographic information of the participants.

	Arm 1 (*n* = 31)	Arm 2 (*n* = 32)
Sex	14 M, 17 F	18 M, 14 F
Age (median, range)	37 (19–62)	41 (22–59)
Race	30 Caucasian 1 Hispanic	32 Caucasian
Onset age (median, range)	31 (14–54)	35 (20–51)

**Table 2:** Efficacy secondary end points at T12 and at T16.

	T12		T16	
	Arm 1	Arm 2	Arm 1	Arm 2
PASI 50	23/25 (92%)	13/24 (54%)	22/25 (88%)	11/24 (46%)
PASI 75	12/25 (48%)	3/24 (12%)	11/25 (44%)	2/24 (8%)
PASI 90	5/25 (20%)	1/24 (4%)	2/25 (8%)	0/24 (0%)
PASI 100	3/25 (12%)	1/24 (4%)	1/25 (4%)	0/24 (0%)
